# Clinical measures of static foot posture do not agree

**DOI:** 10.1186/s13047-016-0180-3

**Published:** 2016-12-01

**Authors:** Ben Langley, Mary Cramp, Stewart C. Morrison

**Affiliations:** 1 Sport and Physical Activity, Edge Hill University, St Helens Road, Lancashire, L39 4QP UK; 2 Centre of Health and Clinical Research, University of the West of England, Bristol, UK; 3 School of Health Sciences, University of Brighton, Brighton, UK

**Keywords:** Morphology, pes planus, pes cavus, Foot classification, Agreement

## Abstract

**Background:**

The aim of this study was to determine the level of agreement between common clinical foot classification measures.

**Methods:**

Static foot assessment was undertaken using the Foot Posture Index (FPI-6), rearfoot angle (RFA), medial longitudinal arch angle (MLAA) and navicular drop (ND) in 30 participants (29 ± 6 years, 1.72 ± 0.08 m, 75 ± 18 kg). The right foot was measured on two occasions by one rater within the same test environment. Agreement between the test sessions was initially determined for each measure using the Weighted Kappa. Agreement between the measures was determined using Fleiss Kappa.

**Results:**

Foot classification across the two test occasions was almost perfect for MLAA (Kw = .92) and FPI-6 (Kw = .92), moderate for RFA (Kw = .60) and fair for ND (Kw = .40) for comparison within the measures. Overall agreement between the measures for foot classification was moderate (Kf = .58).

**Conclusion:**

The findings reported in this study highlight discrepancies between the chosen foot classification measures. The FPI-6 was a reliable multi-planar measure whereas navicular drop emerged as an unreliable measure with only fair agreement across test sessions. The use of this measure for foot assessment is discouraged. The lack of strong consensus between measures for foot classification underpins the need for a consensus on appropriate clinical measures of foot structure.

## Background

Static foot assessment is a common approach in clinical practice for classifying foot type with a view to identifying possible aetiological factors relating to injury and prescribing therapeutic interventions [1, 2]. This approach is underpinned by a contextual model of the foot whereby structural alignment, or position of the foot, is used to infer characteristics of dynamic foot function, and theoretically establish injury mechanisms leading to pathology [3–5]. This model of foot function is primarily derived from the work of Root et al [6, 7] who proposed static assessment measures to enable clinicians to identify deviations from an ideological ‘normal’ foot. A lack of empirical evidence and concerns with the reliability [8] and validity [9, 10] of this work have led to moves away from this approach and to the development of new foot function paradigms [11, 12]. However, despite these more contemporary approaches, the premise of being able to categorise the foot based upon its anatomical characteristics remains appealing and thus static foot assessment remains common. As such numerous foot classification measures have been developed over the past three decades [1, 13, 14]. The majority of these measures, whether they be anthropometric (rearfoot angle (RFA), medial longitudinal arch angle (MLAA), navicular drop (ND)), footprint (arch index, malleolar valgus index) or radiographic measures typically provide only a uni-planar assessment of foot posture. In contrast, the Foot Posture Index [13] (FPI-6) is a multi-planar tool, that combines sagittal, frontal and transverse plane assessments of the foot, that has gained popularity over the past decade.

While there is a plethora of literature exploring the reliability of different foot classification measures there has been little work exploring the level of agreement between different measures. The validity of common static foot measures, specifically the FPI-6, navicular height and Arch Index, was reported in a cohort of older adults [15]. Moderate to strong correlations between clinical measures were reported, with normalised navicular height and FPI-6 demonstrating the highest association (*r* = -.74). Similarly, significant associations (*p* ≤ .01) and moderate to strong correlations (*r* = .42) were reported for clinical and radiographic measurement. These findings are supported by the work of Murley et al., [14] who reported moderate to strong (*r* = .24 - .70) relationships between clinical and radiographic measures. Further work [16] looking at the association between footprint indices (malleolar valgus index and arch index) and navicular measures (navicular drift and drop) reported significant correlations between malleolar valgus index and ND in single (*r* = .61, *p* < .001) and bipedal stance (*r* = .66, *p* < .001). Significant correlations were also reported between Arch Index and navicular drift during single leg stance (*r* = .43, *p* = .029). These studies have all included footprint based tools and the majority have included radiographic measures. The use of footprint indices is contentious [17] due to a lack construct validity [18, 19], while the use of radiographic measures necessitates specialised equipment and exposure to radiation. Furthermore, all of the cited studies have explored the relationship between raw scores, rather than the agreement between measures in relation to which category the foot is classified into. As such the studies offer little indication of the agreement across the measures due to differences in cut off points for foot classification between different metrics. Assessment of the level of agreement would shed light on the consistency with which the foot is classified based on different measures and to the extent to which different foot classification measures are analogous. Information of this kind may in turn help to develop a more standardised approach to static foot classification.

Without doubt access to simple, quick and safe methods to assess the foot is important [20] but, given the number of measures available, there is a need to explore current measures to ensure that the appropriate techniques are used. Consistent, credible and standardised measures are fundamental to informing practitioners involved in foot assessment and care delivery. Equally, the varied use of clinical measures in research studies challenges the pooling and systematic analysis of research data and translation of research findings into clinical practice. Establishing agreement between common measures will help inform debate about the suitability of current measures and ultimately encourage a more standardised approach to clinical practice. Therefore, the aim of this study was to determine the level of agreement between commonly used measures of foot classification.

## Method

### Participants

A convenience sample of 30 participants (29 ± 6 years, 1.72 ± 0.08 m, 75 ± 18 kg) was recruited from staff and students at the institution conducting the study. All participants were asymptomatic and free from injury and any known or visible skeletal abnormality that may have altered foot structure. Institutional ethical approval was granted prior to data collection. All participants provided written informed consent.

### Static foot assessment measures

Our work focused on static foot measures commonly used in our clinical laboratory, specifically FPI-6, ND, RFA, and MLAA. This was a pragmatic decision, based on measures that the team have experience using and a review of the foot classification literature. The right foot was assessed for all participants, only one foot was assessed due to the conceptual and statistical concerns about pooling data from both feet highlighted by Menz [21]. One investigator with three years experience of static foot assessment conducted all testing (BL), with a research assistant recording test scores to help blind the rater and minimise bias within the data. Participants were tested on two occasions within the same test session, with at least ten minutes rest period between measures. Participants were asked to assume a relaxed standing position in double limb support, looking straight ahead with their arms by their sides. The order of testing was consistent throughout the study and measures were conducted in the following order: FPI-6, ND, RFA, and MLAA. Skin markings made on the foot and shank for the ND, RFA and MLAA measures were removed between test and retest.

The FPI-6 was conducted following a standard protocol [22]. Talar head congruency, lateral malleoli curvature, calcaneal inversion/eversion, talonavicular bulging, medial longitudinal arch congruency and forefoot to rearfoot abduction/adduction were measured. Each component was scored on a scale ranging from -2 to +2 and the cumulative score used to define foot type. Foot type was classified according to normative values with scores of ≥ 8 representing a pronated foot type, 0 to 5 a neutral foot and ≤ -1 a supinated foot [23].

**Fig. 1:**
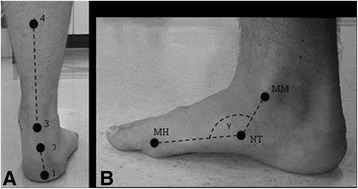
**a** Anatomical locations for rearfoot angle calculation, 1 = base of calcaneus, 2 = Achilles tendon attachment, 3 = centre of Achilles tendon at the height of the medial malleolus and 4 = centre of the posterior aspect of the shank 15 cm above marker 3. **b** Anatomical landmarks used to calculate the MLAA; MM = medial malleolus, NT = navicular tuberosity, MH = first metatarsal head and γ = MLAA

The RFA was measured in accordance with Jonson and Gross [24]. Briefly, four locations were palpated and marked using a skin marker pen (Fig. 1a). These were: (1) the base of the calcaneus; (2) the Achilles tendon attachment; (3) the centre of the Achilles tendon at the height of the medial malleoli; (4.) the centre of the posterior aspect of the shank 15 cm above marker three. The RFA was measured using a goniometer. The arms of the goniometer were aligned with the line connecting marker one and two (line 1) and the other arm with the lines connecting marker three and four (line 2). The RFA was measured as the acute angle between the projection of line one and line two. RFA ≥ 5° valgus represented a pronated foot type, 4° valgus to 4° varus a neutral foot and ≥ 5° varus a supinated foot [24].

For the MLAA, the midpoint of the medial malleolus, the most prominent aspect of the navicular tuberosity and the most medial prominence of the first metatarsal head were palpated and marked using a skin marker pen (see Fig. 1b) [12]. The MLAA was measured using a goniometer with the centre of the goniometer aligned with the navicular mark and the arms aligned to connect the navicular mark with the medial malleolus and first metatarsal head markings. The obtuse angle was recorded as the MLAA. MLAA < 130° represented a pronated foot type, 130° to 150° a neutral foot type and > 150° a supinated foot type [12].

ND was determined following the protocol of Brody [25]. Initially the most prominent aspect of the navicular tuberosity was palpated and marked with a skin marker pen. A piece of card (14.8 x 4.2 cm) was placed next to the medial aspect of the foot and the height of the navicular in a relaxed standing position marked on the card. The foot was then manipulated into subtalar joint neutral as determined by congruence of the talar head, and the process outlined above repeated. ND was recorded as the difference in navicular height between STJN and relaxed standing. ND > 9 mm represented a pronated foot type, 5 to 9 mm a neutral foot and < 5 mm a supinated foot [23].

### Statistical analysis

All data analysis was conducted in Microsoft Excel 2007 and SPSS 20 (IBM, Armonk, NY, USA). Prior to statistical analysis raw FPI-6 scores were converted into logit values in line with Keenan et al., [26]. Data were initially tested for normality using the Shapiro-Wilk test. All data were normally distributed, apart from the ND data. Where data met parametric assumptions Intraclass Correlation Coefficients (ICC_(3,1)_) were used to determine intra-rater reliability. ICC statistics were interpreted as follows; < 0.2 = slight, 0.21–0.4 = fair, 0.41–0.6 = moderate, 0.61–0.8 = substantial and > 0.8 = almost perfect reliability [27]. Where data violated parametric assumptions a weighted Kappa (kw) was used to determine intra-rater reliability. Weighted Kappa (*K*_*w*_) was also used to determine within-measure agreement for foot classification group across test and retest sessions, with weights applied based on a quadratic function. Weights were calculated using equation 1, with the pronated, neutral and supinated categories coded as 1, 2 and 3 respectively.

$$ \mathrm{Weight}={\left({i}_n - {j}_n\right)}^2 $$

Where;

i = row

j = column in test retest matrix

Weighting meant that larger discrepancies between test and retest foot classification groupings received higher weightings thus reducing the Kappa statistic accordingly. To determine between-measure agreement the Fleiss Kappa (*K*_*f*_) was used. The data for each foot classification measure was averaged over the test and retest measures, with the participants foot then classified based upon this average score. Kappa statistics were interpreted as follows; < 0.4 = fair, 0.41–0.6 = moderate, 0.61–0.8 = substantial and > 0.8 = excellent agreement [27].

## Results

Test score, intra-rater reliability and within-measure classification agreement are displayed in Table 1. The intra-rater reliability of the FPI-6 (*ICC*_*(3,1)*_ = .93), RFA (*ICC*_*(3, 1)*_ = .93) and MLAA (*ICC*_*(3, 1)*_ = .91) were almost perfect. ND demonstrated fair reliability (*K*_*w*_ = .4). The level of agreement for foot classification based on each measure across the two test sessions was almost perfect for the FPI-6 (*K*_*w*_ = .92) and MLAA (*K*_*w*_ = .92), moderate for the RFA (*K*_*w*_ = .6) and fair for ND (*K*_*w*_ = .4) (Table 1).

**Table 1:** Test scores (mean (SD)), intra-rater reliability and agreement for the foot classification measures

	Scores		Intra-rater reliability (ICC)	Classification Agreement (*Kw*)
	Test	Retest		
FPI-6	4 (4)^a^	3 (4)^a^	.93	.92
RFA (°)	-3 (3)	- 3 (3)	.93	.60
MLAA (°)	136 (10)	136 (9)	.91	.92
ND (mm)	7 (3)	6 (3)	.40^b^	.40

- represents valgus for RFA

^a^Un-transformed scores

^b^Weighted Kappa

The number of participants classified as having pronated, neutral or supinated feet and the between-measure agreement are detailed in Table 2. Using the FPI-6, 53% of participants were classified as having a neutral foot type, 40% a pronated foot type and 7% a supinated foot type. With the MLAA, 73% of participants had a neutral foot type, 20% of participants a pronated foot type and 7% a supinated foot type. When using the RFA 33% of participants had a neutral foot type, 67% a pronated foot type and 0% had a supinated foot type. Seventy three percent of participants were classified as having a neutral foot type using ND, with 17% a pronated foot type and 10% a supinated foot type. There was moderate agreement (*K*_*f*_ = .58) between the foot classification measures.

**Table 2:** Number of participants classified as having pronated, neutral and supinated feet by each of the static foot classification measures and Fleiss Kappa statistic (*K*_*f*_)

	Pronated	Neutral	Supinated
FPI-6	5	23	2
RFA	10	20	0
MLAA	6	22	2
ND	5	22	3
*K*_*f*_	.58		

FPI-6 = foot posture index, RFA = rearfoot angle, MLAA = medial longitudinal arch angle, ND = navicular drop, *Kf* = Fleiss Kappa statistic

## Discussion

Static foot assessment is commonly undertaken to inform clinical management to identify possible aetiological factors of injury and prescription of therapeutic intervention(s), such as foot orthoses [1, 2]. Consistent, credible and standardised foot measures are key to informing clinical decision making but inconsistencies with the measures and outcome scores pose challenges for practitioners. The aim of this study was to determine the level of agreement between commonly used foot classification measures. Initial within-measure agreement for foot classification was based on the test and retest classification score for each measure. The FPI-6 and MLAA were the most consistent methods for classifying the foot (*Kw* = .92) across two sessions whilst the RFA (*K*_*w*_ = .6) was lower, but with moderate agreement between test sessions. In contrast, ND was the least consistent measure for classifying the foot (*K*_*w*_ = .4) across sessions. The assessment of ND has gained popularity as a simple and quick clinical measure [28] despite conflicting opinion on the reliability of the measure [28, 29]. The findings from this study highlight concerns about the use of the measure as a stand-alone test for foot classification, and re-iterate concerns about the reliability of the measure. The intricacies with navicular tuberosity and sub-talar joint palpation are factors which pose challenges and, as an independent measure, the findings from this study suggest that patients may be misclassified with this measurement and as such the purpose of the measure is challenged.

Agreement between measures for classifying participants’ feet was moderate (*K*_*f*_ *= .58*). This confirms that the measures did not classify participants’ feet into consistent categories (pronated, neutral and supinated). The level of agreement reported in this research is lower than previous studies [15, 16] but this disparity is unsurprising given that the studies have used different measures to classify foot structure, and different approaches to statistical analysis have been undertaken. Due to the different constructs considered by each of the measures in this study our finding may not be surprising but, nevertheless, this remains a concern as the measures purport to classify the foot into three common categories. The moderate level of agreement reported in this study may be explained by the fact that the three dimensional nature of foot structure cannot be represented by a single, uni-planar measure. Our findings re-iterate current opinion that static foot measures are of limited clinical value [20, 30] and that appropriate cut off boundaries for foot classification using reliable measures is required to increase the consistency with which the foot is classified. Furthermore, the limited agreement across measures suggests that the pooling of data across studies using different foot classification tools should be undertaken with caution, as the measures tested are not analogous in the manner in which they classify the foot. One factor that would influence the level of agreement between the measures reported is the classification boundaries used to categories the foot into pronated, neutral and supinated groupings for each measure. The boundaries used within this study for each measure are consistent with those commonly reported within the literature [12, 21, 23, 24]. However, only the cut off boundaries for the FPI-6 are clearly based on normative data. Thus future work is required to determine normative values for the interpretation of static foot classification measures and also a better understanding of how static measures relate to dynamic function and injury.

Based on the data presented in this study, it is our opinion that navicular drop is not an acceptable measure for characterising the foot. Individual measures of foot dimensions (such as navicular drop) may be useful for clinical assessment of specific anatomical sites but it is important to take into account reported findings about the validity, reliability and responsiveness of the measures. The MLAA emerged as the most robust of the uni-planar measures with a higher level of reliability, good agreement within measure for foot classification and broader foot classification boundaries The FPI-6 was the only multi-planar measure used in this study which demonstrated excellent reliability and agreement across sessions. This measure appears to be a robust and reliable means of static foot assessment and offers a more valid approach to assessing static foot structure.

There are some limitations to the work that must be acknowledged. There was a short time frame between test and retest measurements which may have led to a learning or memory effect. To reduce the potential learning or memory effects, a second rater was used to record all scores in an attempt to blind the primary rater to the measured scores. The similarities between the reliability coefficients reported within the study and those previously reported within the literature, where larger time frames between test and retest measurements have been utilised, suggest that no obvious learning effects took place. An additional limitation of the work was the sample recruited into the study. The participants involved in this work were healthy and free from pathology which may limit the external validity of the findings. The recruitment of a healthy population is also likely to have influenced the reliability coefficients reported within this study. It is acknowledged that reliability is a product of a number of factors including the participants, assessor, measure and environment. As such changes in any one of these factors are likely to alter the reliability of the measures reported within this work. We also acknowledge that there are a number of measures that we have not been able to consider in our study.

## Conclusion

Static foot assessment is commonly conducted in clinical practice and the findings reported in this study highlighted moderate agreement between measures for foot classification. These findings highlight the need to carefully consider the clinical measures used for foot classification and suggests that measures should not be used in isolation. Navicular drop emerged as an unreliable measure with only fair agreement across test sessions and use of this measure is discouraged. The FPI-6 is a multi-planar measure which was found to be a reliable measure for evaluating static foot position. It remains important for clinicians and researchers to consider the role of uni-planar measures in the assessment of the foot. The lack of strong consensus between measures for foot classification underpins the need for a consensus on appropriate clinical measures of foot structure.

## Abbreviations

FPI-6: Foot Posture Index
ICC: Intraclass correlation coefficient
MLAA: Medial Longitudinal Arch Angle
ND: Navicular Drop
RFA: Rearfoot Angle
